# Integrated analysis of morphological traits and salinity stress-related gene expression in alfalfa (*Medicago sativa* L.) from Türkiye under magnesium sulfate and calcium chloride treatments

**DOI:** 10.3389/fpls.2026.1764060

**Published:** 2026-05-07

**Authors:** Muhammed İkbal Çatal, Seda Mesci

**Affiliations:** 1 Department of Field Crops, Faculty of Agriculture, Recep Tayyip Erdoğan University, Rize, Türkiye; 2 Project Coordination and Guidance Office, Rectorate, Hitit University, Çorum, Türkiye; 3 Food Safety, Agricultural Application and Research Center, Hitit University, Çorum, Türkiye

**Keywords:** abiotic stress, alfalfa, calcium chloride, gene expression, magnesium sulfate, *Medicago sativa*, salt stress

## Abstract

*Medicago sativa* L. (alfalfa) has been cultivated worldwide for thousands of years, including in Türkiye, and is recognized as an important forage crop due to its high nutritional value, yield potential, and adaptability to diverse environmental conditions. Therefore, the development of cultivars adapted to different ecological regions and the evaluation of their performance are essential for sustainable forage production. This study examined the effects of salt stress on alfalfa growth, morphology, and gene expression. Seven alfalfa cultivars from Türkiye (Kaval, Kayseri, Elçi, Sunter, Özpınar, Bilensoy-Covered/Uncovered) were treated with aqueous solutions of Magnesium Sulfate (30 mM) and Calcium Chloride (30 mM). Morphological traits—including plant height, stem diameter, node number, internode length, leaf number, leaflet length, and leaflet width—were measured prior to harvest. In addition, RNA isolation, cDNA synthesis, and RT-qPCR analyses were conducted to assess the expression levels of salt stress–related genes. A decreasing trend in the expression of salt stress-related genes was observed, particularly *GR* was observed in the cultivars Kaval, Kayseri, Özpınar, and Bilensoy in response to both magnesium sulfate and calcium chloride treatments. The correlation analysis (r = 0.81) suggests a strong associative relationship between *GR* expression and morphological performance, rather than a direct functional linkage. These findings should be interpreted as biological trends reflecting the coordinated response of the antioxidant system and plant growth under salinity stress. The results may provide a theoretical basis for the cultivation of alfalfa in soils high in MgSO_4_ and CaCl_2_.

## 1 Introduction

The genus *Medicago* consists of approximately 80 to 100 species, among which common alfalfa (*Medicago sativa L.*) is the most widely cultivated both globally and in Türkiye. The domestication of this species dates back approximately 8–9 millennia. Recognized as a crucial leguminous forage crop, alfalfa is highly valued for its rich nutritional profile, substantial productivity, and adaptability across diverse ecological zones (Açıkgöz, 2001; Tan, 2018; Zubair et al., 2017; Guo et al., 2024a; Zhang and Wang, 2025). Notably, compared to other forage crops, alfalfa exhibits superior performance in well-lit, warm, and arid conditions, benefiting from a deep root system that confers drought resistance. It can be successfully grown under rainfed conditions in regions with 350–450 mm of annual precipitation, allowing for multiple harvests per year (Açıkgöz, 2001; Tan, 2018; Zeyliger et al., 2023; Begna et al., 2025).

Often recognized as the “queen of forage crops” due to its broad adaptability (Tekeli and Ateş, 2011; Guo et al., 2024b; Zhang and Wang, 2025), and versatile applications alongside high yields, common alfalfa’s capacity to thrive in a wide range of climatic and soil conditions has facilitated its cultivation across nearly all of Türkiye (Anonymous, 1999). As a significant source of protein, minerals, and vitamins, it provides highly nutritious livestock feed, enabling multiple cuttings annually depending on environmental factors (Appiah et al., 2024; Zhang and Wang, 2025). Furthermore, its perennial nature, rapid growth, and harvestability contribute to its strong competitive ability against weeds (Uygur, 1991; Ma et al., 2023a). Given its increasing cultivation in Türkiye, research focusing on developing new, region-specific varieties and evaluating their adaptation is crucial, alongside identifying pasture-type alfalfa for rangeland improvement (Kır and Soya, 2008; Wang and Zhang, 2023). While alfalfa offers high yield potential and nutritious forage with low cellulose content, production challenges exist, with studies indicating shifts in leaf-to-stem ratio and protein content as plant height increases (Manga, 1981; Akbari and Avcıoğlu, 1994; Aydın et al., 1994; Tahtacıoğlu et al., 1994; Şengül and Tahtacıoğlu, 1996; Putnam, 2021; Bhandari et al., 2023; Hadidi et al., 2023).

Abiotic stresses adversely impact plant survival and development, consequently reducing crop yield. Molecular regulatory mechanisms involving gene and enzymes such as catalase (*CAT*), glutathione reductase (*GR*), superoxide dismutase (*SOD*), phytochelatins (*PCS*), peroxidase (*POD*), and zinc-regulated transporters (*ZIP*) may play active roles in modulating plant responses to salinity, drought, and cold stress in various plant species (Wang et al., 2004; Zhai et al., 2023; Ismaiel et al., 2024; Zhang et al., 2024a; Yang et al., 2025).

Salinity-induced stress remains a pressing global issue that significantly hampers agricultural productivity and poses a serious threat to food security (Sundar et al., 2025). Few studies have examined the expression and activity of salt stress-related genes and proteins (e.g., *CAT, GR, SOD, PCS, POD*, and *ZIP*) in alfalfa (Rahman et al., 2020; Guiza et al., 2022; Hou et al., 2022; Li et al., 2022a, Li et al., 2022b; Ma et al., 2023b; Pacheco-Insausti et al., 2023; Zhang et al., 2024b). Recent studies have highlighted the involvement of *ZIP* transporters in maintaining zinc homeostasis and ionic equilibrium under salt or oxidative stress conditions (Ajeesh Krishna et al., 2020; Shao et al., 2023).

Salinity research predominantly focuses on Na^+^ salts (e.g., NaCl). However, large areas of saline-affected agricultural lands in Türkiye, particularly in the Central and Eastern Anatolian regions, are characterized by high concentrations of divalent cations, specifically Magnesium (Mg^2+^) and Calcium (Ca^2+^), often leading to saline-sodic or MgSO_4_ -dominant salinity. These salts exert distinct physiological impacts compared to Na^+^ salts; Ca^2+^ is crucial for maintaining membrane integrity and mitigating Na^+^ toxicity, while excess Mg^2+^ can interfere with K^+^ uptake and enzyme activities. While NaCl-induced stress is characterized by Na+ toxicity and osmotic shock, MgSO_4_ and CaCl_2_ introduce challenges related to divalent cations. Excess Mg2+ can antagonize K+ uptake and disrupt enzymatic functions, while high concentrations of CaCl2 can lead to Cl- toxicity and interfere with the absorption of essential nutrients like P and Mg. By focusing on these divalent salts, this study addresses the specific soil chemistry challenges of Central and Eastern Anatolia, providing a broader understanding of salt stress beyond the common monovalent ion paradigms. Therefore, the use of MgSO_4_ and CaCl_2_ in this study is agronomically relevant to the specific soil chemistry challenges facing Turkish alfalfa cultivation and offers novel insights into cultivar-specific regulatory response mechanisms to divalent cation–induced stress.

Considering the well-established importance and widespread cultivation of common alfalfa in Türkiye, and the recognized need to improve its resilience under diverse environmental stressors, this study focuses on evaluating the impact of salinity on different alfalfa genotypes. To address this challenge, we hypothesize that exogenous application of magnesium sulfate (MgSO_4_) and calcium chloride (CaCl_2_) may differentially influence the morphological traits and the expression of key salt stress-responsive genes across seven distinct alfalfa cultivars. By investigating these responses, we aim to identify potential varietal differences in stress-related gene regulatory mechanisms and to provide a foundation for developing targeted strategies to improve alfalfa productivity in saline-affected regions of Türkiye.

The objective was to determine how these treatments influence plant height, stem diameter, node number, internode length, leaf number, leaflet length, and leaflet width, and to elucidate the molecular mechanisms underlying alfalfa’s response to salt stress by analyzing the gene expression of *GR*, *PCS*, *SOD*, and *ZIP*. Notably, this research represents one of the first integrative studies to simultaneously assess both morphological and transcriptional responses to MgSO_4_ and CaCl_2_*-*induced salinity in alfalfa. The findings revealed cultivar-specific stress-related gene regulatory mechanisms and identified potential genetic and phenotypic targets for improving alfalfa performance under saline conditions.

## 2 Materials and methods

### 2.1 Plant material and experimental design in alfalfa (*Medicago sativa* L.)

This study was conducted in a greenhouse located in the Pazar district of Rize province, Türkiye. Seven alfalfa cultivars - Kaval, Kayseri, Elçi, Sunter, Bilensoy-covered and Bilensoy-uncovered seeds (Melih Tarım, commercial company) and Özpınar (Aegean Agricultural Research Institute) were sown in pots containing peat. After four weeks of emergence, the plants were treated with aqueous solutions of Magnesium Sulfate heptahydrate (MgSO_4_-7H_2_O) and Calcium Chloride (CaCl_2_) at a concentration of 30 millimolar (30 mM). The salt treatments were applied as a single, one-time application by distributing an equal volume of solution to each pot without disturbing the soil. This concentration resulted in an approximate root-zone Electrical Conductivity (EC) of 3.0-4.0 dS/m, indicative of moderate salt stress.

The experiment was arranged in a split-plot design with three replicates (blocks). The main plots consisted of the seven alfalfa varieties, and the sub-plots consisted of the three treatments (Control, Magnesium Sulfate [30 mM], and Calcium Chloride [30 mM]). All morphological data were subjected to Two-Way Analysis of Variance (ANOVA) using the 63 individual measurements. However, for presenting the mean data in the tables and conducting the correlation analysis (**Table 1**), the mean value of the three biological replicates for each Cultivar*Treatment combination was calculated, resulting in n=21 data points (7 Cultivars * 3 Treatments) used for the correlation analysis. The analysis aims to capture the overall physiological trend of genotype-treatment interactions rather than individual replicate variance.

**Table 1:** Correlation matrix and p-values among morphological traits in alfalfa cultivars.

Correlation probability	Plant height	Stem diameter	Node number	Internode length	Number of leaves	Length of leaflets	Width of leaflets
Plant height p-value	1.0000	0.5771	0.4975	0.6918	0.5528	0.7643	0.5262
	<.0001**	<.0001**	<.0001**	<.0001	<.0001**	<.0001**	<.0001**
Stem diameter p-value	0.5771	1.0000	0.2601	0.3473	0.1884	0.4502	0.3193
	<.0001**	<.0001**	0.0396*	0.0053	0.1392	0.0002**	0.0108*
Node number p-value	0.4975	0.2601	1.0000	0.3951	0.4906	0.4849	0.2089
	<.0001**	0.0396*	<.0001**	0.0014	<.0001**	<.0001**	0.1003
Internode length p-value	0.6918	0.3473	0.3951	1.0000	0.4261	0.6336	0.3466
	<.0001**	0.0053**	0.0014**	<.0001	0.0005**	<.0001**	0.0054**
Number of leaves p-value	0.5528	0.1884	0.4906	0.4261	1.0000	0.4030	0.2510
	<.0001**	0.1392	<.0001**	0.0005	<.0001**	0.0011**	0.0472*
Length of leaflets p-value	0.7643	0.4502	0.4849	0.6336	0.4030	1.0000	0.3578
	<.0001**	0.0002**	<.0001**	<.0001	0.0011**	<.0001**	0.0040**
Width of leaflets p-value	0.5262	0.3193	0.2089	0.3466	0.2510	0.3578	1.0000
	<.0001**	0.0108*	0.1003	0.0054	0.0472*	0.0040**	<.0001**

**p<0.01, *p<0.05, Sample size for correlation analysis: n=21.

Before harvest, several morphological characteristics were measured in the early vegetative stage (8 weeks after planting): plant height (cm), stem diameter (mm), node number (nodes/plant), internode length (mm), number of leaves (number/plant), length of leaflets (mm), and width of leaflets (mm).

The data obtained from the morphological measurements were subjected to Two-Way Analysis of Variance (ANOVA) for a Split-Plot Design using JMP 13.2 statistical software. Significant differences among means were grouped according to Tukey’s Honestly Significant Difference (HSD) test at a significance level of p<0.05.

### 2.2 RNA isolation in alfalfa (*Medicago sativa* L.)

A total of 300 mg of *Medicago sativa* plant tissue from each sample (1-21) was ground in a porcelain mortar cooled with liquid nitrogen. The ground tissue was homogenized with 500 µL of lysis buffer and transferred into 1.5 mL microcentrifuge tubes. The mixture was vortexed and incubated at room temperature for 5 minutes. Subsequently, 100 µL of chloroform was added, followed by a 3-minute incubation at room temperature, and centrifugation at 12000 rpm for 15 minutes at 4 °C. The upper aqueous phase was transferred to a new microcentrifuge tube and mixed with 250 µL of isopropanol. The sample was then applied to a purification column placed in a collection tube and centrifuged at room temperature. Washing steps were performed sequentially using wash buffers I and II. Finally, 50 µL of elution buffer was added to the column, incubated for 1 minute at room temperature, and centrifuged to collect the RNA (Ecotech, Plant Total RNA Kit). Isolated RNA samples were stored at -20 °C. RNA isolation, cDNA synthesis, and qRT-PCR analysis for all samples (1–21) were conducted in single replicates (**Tables 2**, **3**).

**Table 2:** Forward/reverse sequences and melting temperature (TM) of the primers belonging to *Medicago sativa* genes.

Genes	Forward sequences (5’-3’)	Reverse sequences (5’-3’)	TM (°C)
*β-Actin*	CTTCGCGGGCGACGAT	CACATAGGAATCCTTCTGACCCAT	59
*GR*	GTGCTTCGTGGATGTGTTCCAAAG	GTGCTCCAGTCATGCTTCGGATCAG	63
*PCS*	TTTCAAGTATCCTCCTCACTGGGTTC	TTCATCTTTACARCTCACAGTAT	60
*SOD*	TAATTGGCTCCAAAGCATTC	TCTTTTCTTCCCAATGTTGC	53
*ZIP*	CGAGGAAAACCGTTATACGA	GAGAAGGGAAAATCCTTCCA	55

**Table 3:** RNA quality, quantity, and raw Cycle Threshold (Ct) values from qRT-PCR analysis of salt stress-related genes in seven alfalfa cultivars under control and salt stress conditions.

No	Samples (1-21)	RNA amounts (ng/μl)	260/280 ratio (nm)	β-Actin (Ct)	*GR*(Ct)	*PCS* (Ct)	*SOD* (Ct)	*ZIP* (Ct)
1	Control (Kaval)	397.244	2.10	29.49	41.07	27.81	38.74	26.67
2	Magnesium Sulfate (30mM)	101.707	2.15	30.01	36.09	25.8	35.61	26.9
3	Calcium Chloride (30mM)	123.169	2.08	29.75	39.79	31.84	35.82	28.63
4	Control (Kayseri)	110.666	2.17	28.78	40.49	28.29	38.62	28.36
5	Magnesium Sulfate (30mM)	375.972	2.03	28.31	36.03	28.54	36.13	27.1
6	Calcium Chloride (30mM)	125.188	2.05	29.61	38.99	26.26	36.22	29.19
7	Control (Elçi)	77.378	2.31	28.25	39.2	27.87	36.27	29.02
8	Magnesium Sulfate (30mM)	386.482	2.05	29.02	43.4	27.48	35.68	30.09
9	Calcium Chloride (30mM)	268.42	2.15	27.5	40.79	28.27	34.48	27.68
10	Control (Sunter)	181.52	2.11	28.53	30.91	28.78	39.09	24.49
11	Magnesium Sulfate (30mM)	104.314	2.14	28.3	34.54	26.82	38.32	25.61
12	Calcium Chloride (30mM)	184.88	2.06	28.47	34.25	28.54	37.08	26.29
13	Control (Bilensoy-covered)	33.69	2.10	28.44	43.6	29.17	36.13	26.21
14	Magnesium Sulfate (30mM)	209.112	2.09	28.0	36.09	30.77	35.89	26.77
15	Calcium Chloride (30mM)	139.671	2.09	29.58	42.06	27.3	38.47	28.72
16	Control (Özpınar)	66.74	1.97	29.31	42.89	24.55	37.77	27.25
17	Magnesium Sulfate (30mM)	196.881	2.11	27.7	37.57	28.11	37.16	28.52
18	Calcium Chloride (30mM)	169.391	2.15	30.06	41.98	27.35	39.78	29.92
19	Control (Bilensoy-uncovered)	140.587	2.24	29.05	41.94	26.19	34.81	23.49
20	Magnesium Sulfate (30mM)	217.748	2.09	28.97	40.0	27.14	39.55	26.61
21	Calcium Chloride (30mM)	148.023	2.16	29.01	38.03	27.72	36.69	27.29

### 2.3 Preparation of cDNA

Conventional PCR (Thermo Scientific) was performed using a reaction mixture containing 10 µL of total RNA (100 ng), 4 µL of cDNA synthesis kit, and 6 µL of ddH_2_O, following the manufacturer’s protocol. The reaction was carried out at 42 °C for 100 minutes, followed by 5 minutes at 85 °C. The synthesized cDNA products were stored at -20 °C for subsequent qPCR analysis (Ecotech, 5x First Strand cDNA Synthesis Kit). cDNA samples obtained from alfalfa were analyzed using the Ecotech SYBR Green qPCR Assays Kit according to the manufacturer’s protocol, with a Roche LightCycler 96 Real-Time PCR system. Gene sequences were identified via the NCBI database. Primers required for RT-PCR were designed using the NCBI Primer-BLAST tool (https://www.ncbi.nlm.nih.gov/tools/primer-blast). The gene expression levels of salt stress-related (*GR, PCS, SOD*, and *ZIP*) were investigated using the qRT-PCR method. β-Actin was used as a housekeeping reference gene. The forward and reverse primer sequences for alfalfa are listed in **Tables 2** and **3**.

### 2.4 Quantitative RT-PCR analysis of salt stress-responsive expression in alfalfa (*Medicago sativa* L.)

**Figure 1:**
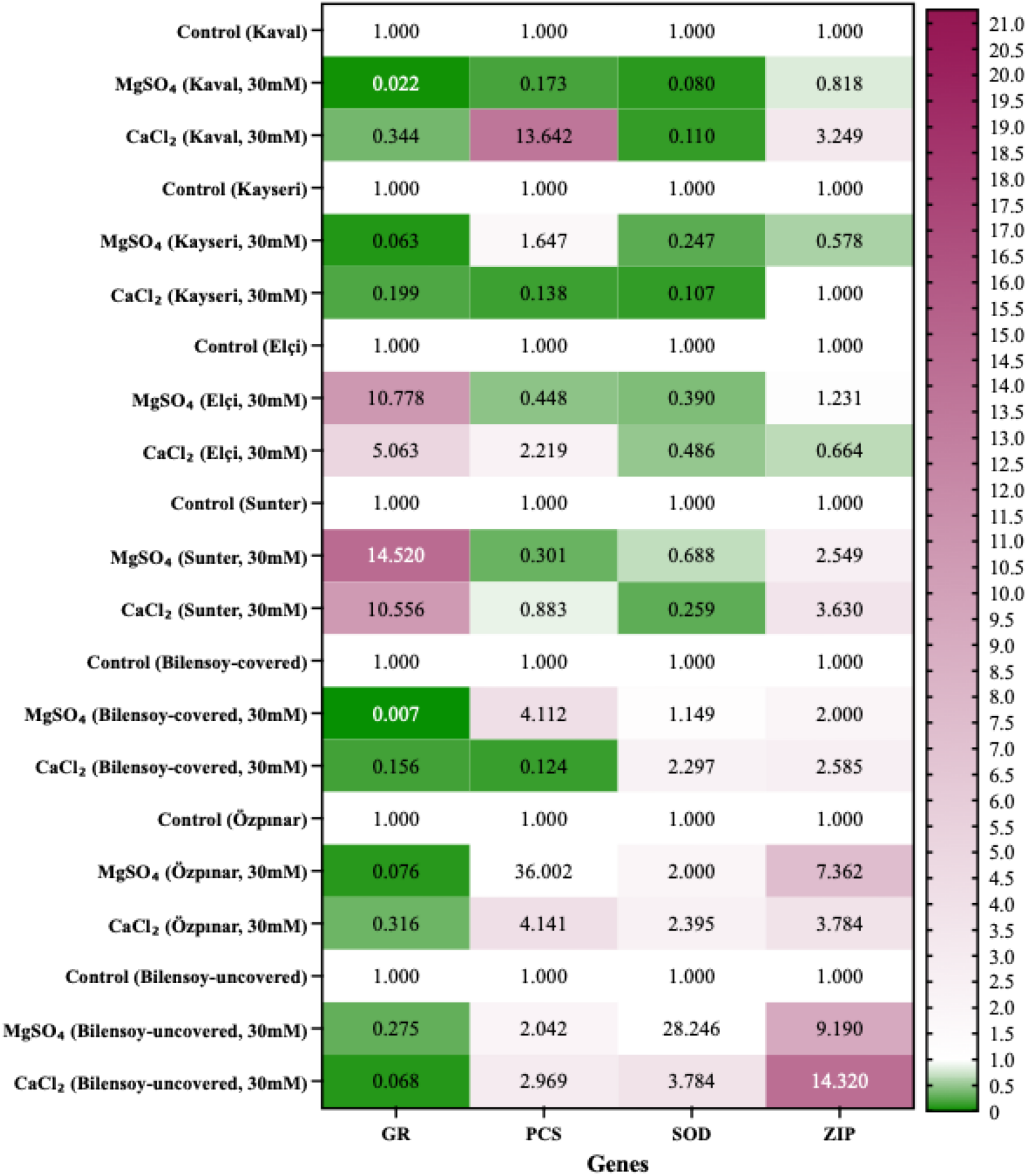
qRT-PCR mRNA gene expression levels of the *GR, PCS, SOD*, and *ZIP* gene in *Medicago sativa* samples (1-21), Control: untreated (Fold change: 1.000).

In qPCR analysis, the cycle threshold (Ct) value is critical for determining the number of amplification cycles required for the fluorescent signal to exceed the background threshold. To evaluate gene expression levels, the 2^−ΔΔCt^ method (ΔCt = Ct_target − Ct_reference/ΔΔCt = ΔCt_treated − ΔCt_control) was applied, following the steps described by Livak and Schmittgen (2001). (**Figure 1**; **Supplementary Figures 1**–**4**; **Table 3**). One-way ANOVA was used to assess the expression levels of salt stress-related genes (*GR*, *PCS*, *SOD*, and *ZIP*) in alfalfa samples (1–21) compared to the control group, using the Prism 9 software.

The stability of the reference gene (β-Actin) was evaluated based on the variation of raw Ct values across all samples. RNA quality and purity were confirmed prior to qRT-PCR analysis, with 260/280 ratios ranging between 1.97 and 2.31. The Ct values of β-Actin showed low variability across control and salt stress treatments and among different cultivars (mean Ct = 28.86 ± 0.73, CV = 2.53%), indicating stable expression under the experimental conditions. Therefore, β-Actin was considered suitable for normalization of qRT-PCR data.

## 3 Results and discussion

### 3.1 Morphological indicators in alfalfa (*Medicago sativa* L.)

**Table 4** presents the morphological measurement results obtained from different alfalfa cultivars under varying salt application treatments. The data from the two-way ANOVA showed a statistically significant effect of the ‘Cultivar’ factor on Plant Height and Node Number (p<0.05 and p<0.01, respectively). The main effect of ‘Treatment’ was *not* significant (ns) for any morphological trait. However, a highly significant cultivar* Treatment interaction (p<0.01) was observed for Plant Height, Internode Length, Length of Leaflets, and Width of Leaflets. This interaction highlights that the effect of salt treatment is highly dependent on the specific alfalfa cultivar.

**Table 4:** Morphological measurement results (Mean ± SD) of seven alfalfa cultivars under control, Calcium Chloride (30 mM), and Magnesium Sulfate (30 mM) treatments.

Sources of variation	df	Plant height	Stem diameter	Node number	Internode length	Number of leaves	Length of leaflets	Width of leaflets
Cultivar	6	F=3.12 P=0.02 *	F=1.98 P=0.09 ns	F=4.86 P=0.01 **	F=2.06 P=0.08 ns	F=1.62 P=0.16 ns	F=2.21 P=0.06 ns	F=0.35 P=0.90 ns
Treatment	2	F=2.68 P=0.08 ns	F=0.43 P=0.65 ns	F=1.63 P=0.21 ns	F=1.88 P=0.17 ns	F=0.26 P=0.77 ns	F=2.74 P=0.08 ns	F=1.00 P=0.38 ns
Cultivar* Treatment	12	F=3.63 P=0.01 **	F=1.42 P=0.19 ns	F=1.56 P=0.14 ns	F=2.77 P=0.01 **	F=1.81 P=0.08 ns	F=3.27 P=0.01 **	F=3.01 P=0.01 **
Error	42	–	–	–	–	–	–	–
CULTIVAR		Plant height (cm)*	Stem diameter (mm)	Node number (nodes/plant)**	Internode length (mm)	Number of leaves (number/plant)	Length of leaflets (mm)	Width of leaflets (mm)
Bilensoy-covered		6.66± 1.90 ab	0.65± 0.14	2.67± 0.71 b	8.69± 7.09	3.89± 0.78	8.12± 2.37	6.13± 2.20
Bilensoy-uncovered		9.46± 3.63 a	0.77± 0.20	3.67± 0.71 ab	13.68± 8.29	4.44± 0.73	11.15± 3.61	6.32± 1.66
Elçi		7.58± 2.98 ab	0.63± 0.13	2.67± 0.50 b	17.22± 10.74	3.89± 0.60	9.49± 2.75	5.92± 0.82
Kaval		6.97± 2.05 ab	0.70± 0.25	4.00± 1.00 a	15.05± 3.61	4.33± 1.12	9.35± 1.74	6.05± 1.36
Kayseri		5.33± 2.00 b	0.56± 0.11	2.89± 0.60 b	9.20± 2.93	3.33± 1.00	8.24± 1.51	5.89± 1.21
Özpınar		7.43± 2.17 ab	0.61± 0.18	3.33± 0.71 ab	11.23± 10.41	4.11± 1.17	9.13± 2.48	5.52± 1.42
Sunter		7.40± 3.36 ab	0.53± 0.20	3.22± 0.83 ab	10.98± 7.83	4.00± 0.87	10.15± 3.42	5.94± 1.33
Treatment (millimolar)		Plant height (cm)	Stem diameter (mm)	Node number (nodes/plant)	Internode length (mm)	Number of leaves (number/plant)	Length of leaflets (mm)	Width of leaflets (mm)
Control		6.40± 2.13	0.63± 0.20	3.00± 0.55	10.20± 6.06	3.90± 0.77	8.52± 1.96	5.76± 1.69
Calcium Chloride (30 mM)		7.60± 2.73	0.66± 0.15	3.38± 1.02	12.60± 7.61	4.00± 0.89	9.57± 3.08	6.27± 1.31
Magnesium Sulfate (30 mM)		7.78± 3.30	0.61± 0.20	3.24± 0.89	14.09± 9.71	4.09± 1.14	10.03± 2.86	5.86± 1.26
Cultivar* Treatment (millimolar)		Plant height (cm)**	Stem diameter (mm)	Node number (nodes/plant)	Internode length (mm)**	Number of leaves (number/plant)	Length of leaflets (mm)**	Width of leaflets (mm)**
Bilensoy-covered* Control		7.13± 1.37 ab	0.73± 0.19	2.33± 0.58	6.16± 3.34 b	3.67± 0.58	7.10± 2.80 c	8.48± 2.30 a
Bilensoy-covered *Calcium Chloride (30 mM)		7.50± 2.23 ab	0.63± 0.13	3.00± 1.00	13.01± 10.53 ab	4.33± 1.15	9.60± 2.61 abc	5.38± 0.99 ab
Bilensoy-covered* Magnesium Sulfate (30 mM)		5.33± 1.89 b	0.59± 0.09	2.67± 0.58	6.90± 6.07 b	3.67± 0.58	7.63± 1.64 bc	4.53± 0.38 b
Bilensoy-uncovered*Control		7.40± 1.64 ab	0.66± 0.09	3.00± 0.20	8.59± 2.98 b	4.00± 0.20	9.08± 0.42 abc	4.91± 1.45 ab
Bilensoy-uncovered*Calcium Chloride (30 mM)		7.47± 1.05 ab	0.67± 0.06	3.67± 0.58	11.33± 5.44 ab	4.33± 0.58	8.95± 2.98 abc	6.42± 1.41 ab
Bilensoy-uncovered*Magnesium Sulfate (30 mM)		13.50± 3.50 a	0.97± 0.22	4.33± 0.58	21.13± 10.30 ab	5.00± 1.00	15.41± 1.43 a	7.60± 1.25 b
Elçi*Control		8.17± 2.90 ab	0.72± 0.19	2.67± 0.58	14.92± 5.53 ab	3.67± 0.58	10.15± 2.42 abc	5.69± 0.64 ab
Elçi*Calcium Chloride (30 mM)		4.53± 1.54 b	0.58± 0.05	2.33± 0.58	7.09± 4.60 b	3.67± 0.58	6.94± 1.76 c	6.06± 0.98 ab
Elçi*Magnesium Sulfate (30 mM)		10.03± 1.17 ab	0.59± 0.12	3.00± 0.20	29.66± 3.92 a	4.33± 0.58	11.36± 2.40 abc	5.98± 1.09 ab
Kaval*Control		4.71± 0.12 b	0.75± 0.40	3.33± 0.58	11.82± 2.49 ab	4.00± 1.00	7.79± 1.11 bc	4.79± 0.84 ab
Kaval*Calcium Chloride (30 mM)		8.23± 1.07 ab	0.77± 0.16	4.33± 1.53	18.02± 2.64 ab	3.67± 0.58	10.39± 1.43 abc	7.24± 1.32 ab
Kaval*Magnesium Sulfate (30 mM)		7.97± 2.02 ab	0.57± 0.16	4.33± 0.58	15.30± 3.16 ab	5.33± 1.15	9.86± 1.78 abc	6.13± 0.63 ab
Kayseri*Control		4.90± 1.05 b	0.54± 0.05	3.33± 0.58	9.67± 2.94 ab	4.33± 0.58	8.80± 1.17 abc	5.55± 0.52 ab
Kayseri*Calcium Chloride (30 mM)		6.13± 1.07 b	0.69± 0.16	2.67± 1.53	8.52± 2.64 b	3.00± 0.58	7.52± 1.43 bc	5.78± 1.32 ab
Kayseri*Magnesium Sulfate (30 mM)		4.97± 1.47 b	0.46± 0.08	2.67± 0.58	9.41± 4.67 ab	2.67± 0.58	8.38± 1.36 bc	6.35± 1.50 ab
Özpınar*Control		7.33± 3.51 ab	0.62± 0.13	3.33± 0.58	15.38± 12.15 ab	4.33± 1.53	8.69± 3.52 bc	6.04± 1.92 ab
Özpınar*Calcium Chloride (30 mM)		8.53± 1.72 ab	0.61± 0.26	3.67± 0.58	13.45± 13.80 ab	4.33± 0.58	9.61± 3.18 abc	5.93± 1.54 ab
Özpınar*Magnesium Sulfate (30 mM)		6.43± 0.51 b	0.60± 0.20	3.00± 1.00	4.88± 1.35 b	3.67± 1.53	9.09± 1.20 abc	4.60± 0.25 b
Sunter*Control		5.17± 0.84 b	0.41± 0.15	3.00± 0.20	4.85± 2.08 b	3.33± 0.58	8.04± 0.84 bc	4.85± 1.03 ab
Sunter*Calcium Chloride (30 mM)		10.80± 4.03 ab	0.68± 0.27	4.00± 1.00	16.76± 8.52 ab	4.67± 1.15	13.94± 3.56 ab	7.09± 1.47 ab
Sunter*Magnesium Sulfate (30 mM)		6.23± 3.50 b	0.49± 0.22	2.67± 0.58	11.34± 10.30 ab	4.00± 1.00	8.45± 1.43 bc	5.88± 1.25 ab
Mean		7.26	0.63	3.21	12.30	4.00	9.37	5.96

p < 0.05; ** p < 0.01; ns, not significant. ANOVA was performed based on a Split-Plot Design. df, Degrees of freedom. Data represent the mean ± standard deviation of three biological replicates (n=3) per Cultivar*Treatment combination. Means in the same column followed by the same lowercase letter are not significantly different according to Tukey’s HSD test.

The morphological measurements of the alfalfa cultivar under different treatments revealed significant variations across several parameters. As indicated in **Table 4**, the cultivar exhibited a statistically significant (p < 0.01) effect on node number and plant height. Furthermore, a significant interaction (p < 0.01) between cultivar and treatment was observed for internode length and length of leaflets.

Specifically, within the cultivar comparison, ‘Bilensoy-Uncovered’ displayed the highest plant height (9.46 cm), while ‘Kayseri’ recorded the lowest (5.33 cm). ‘Bilensoy-uncovered’ presented the largest stem diameter (0.77 mm), and ‘Kaval’ the smallest (0.70 mm). Notably, ‘Kaval’ exhibited the highest node number (4.00 nodes/plant), significantly greater than ‘Bilensoy-covered’ (2.67 nodes/plant). The longest internode length was observed in ‘Elçi’ (17.22 mm), whereas ‘Bilensoy-covered’ had the shortest (8.69 mm). The number of leaves ranged from 3.33 in ‘Kayseri’ to 4.44 in ‘Bilensoy-uncovered’. ‘Bilensoy-uncovered’ also presented the longest leaflets (11.15 mm), while ‘Bilensoy-covered’ had the shortest (8.12 mm). The widest leaflets were observed in ‘Bilensoy-covered’ (6.13 mm) and the narrowest in ‘Özpınar’ (5.52 mm).

Regarding the treatment effects, plants treated with Magnesium Sulfate (30 mM) showed the highest plant height (7.78 cm), while the control group had the lowest (6.40 cm). Stem diameter was similar across all treatments. Calcium Chloride (30 mM) resulted in the highest node number (3.38 nodes/plant) and internode length (12.60 mm). The number of leaves was highest in the Magnesium Sulfate treatment (4.09 leaves/plant). Plants treated with Magnesium Sulfate also exhibited the longest leaflets (10.03 mm), and Calcium Chloride resulted in the widest leaflets (6.27 mm).

The significant interaction between cultivar and treatment for internode length and plant height suggests that the treatment effects were cultivar specific. The application of salt stress generally resulted in a reduction in most morphological parameters across all cultivars. However, the magnitude of this reduction varied depending on the cultivar and the specific salt treatment. For instance, ‘Bilensoy-covered’ treated with 30 mM Calcium Chloride showed a plant height of 7.50 cm, while ‘Elçi’ treated with 30 mM Magnesium Sulfate displayed a plant height of 10.03 cm, demonstrating the differential responses of cultivars to the specific salt type.

Interestingly, certain cultivars exhibited a degree of potential response pattern or even enhancement in specific traits under specific salt treatments. For example, ‘Bilensoy-uncovered’ treated with Calcium Chloride (30 mM) showed a relatively high leaflet length (8.95 mm) and width (6.42 mm) compared to other stressed cultivars. Similarly, ‘Elçi’+Magnesium Sulfate (30 mM) demonstrated the longest internodes (29.66 mm) among all treated groups.

In **Table 4**, the mean values obtained from the current pot experiment under greenhouse conditions revealed an average plant height of 7.26 cm, a stem diameter of 0.63 mm, 3.21 nodes per plant, an internode length of 12.30 mm, 4.00 leaves per plant, a leaflet length of 9.37 mm, and a leaflet width of 5.96 mm across all cultivars and treatments. These findings offer a baseline understanding of the early vegetative growth characteristics of the studied alfalfa cultivars under controlled, albeit confined, conditions.

These findings highlight the differential responses of alfalfa cultivars to salinity stress and suggest the potential for identifying cultivars with inherent tolerance or the possibility of mitigating the negative effects of salinity through specific nutrient applications. Further analysis is warranted to elucidate the underlying mechanisms of these varied responses.

**Figure 2:**
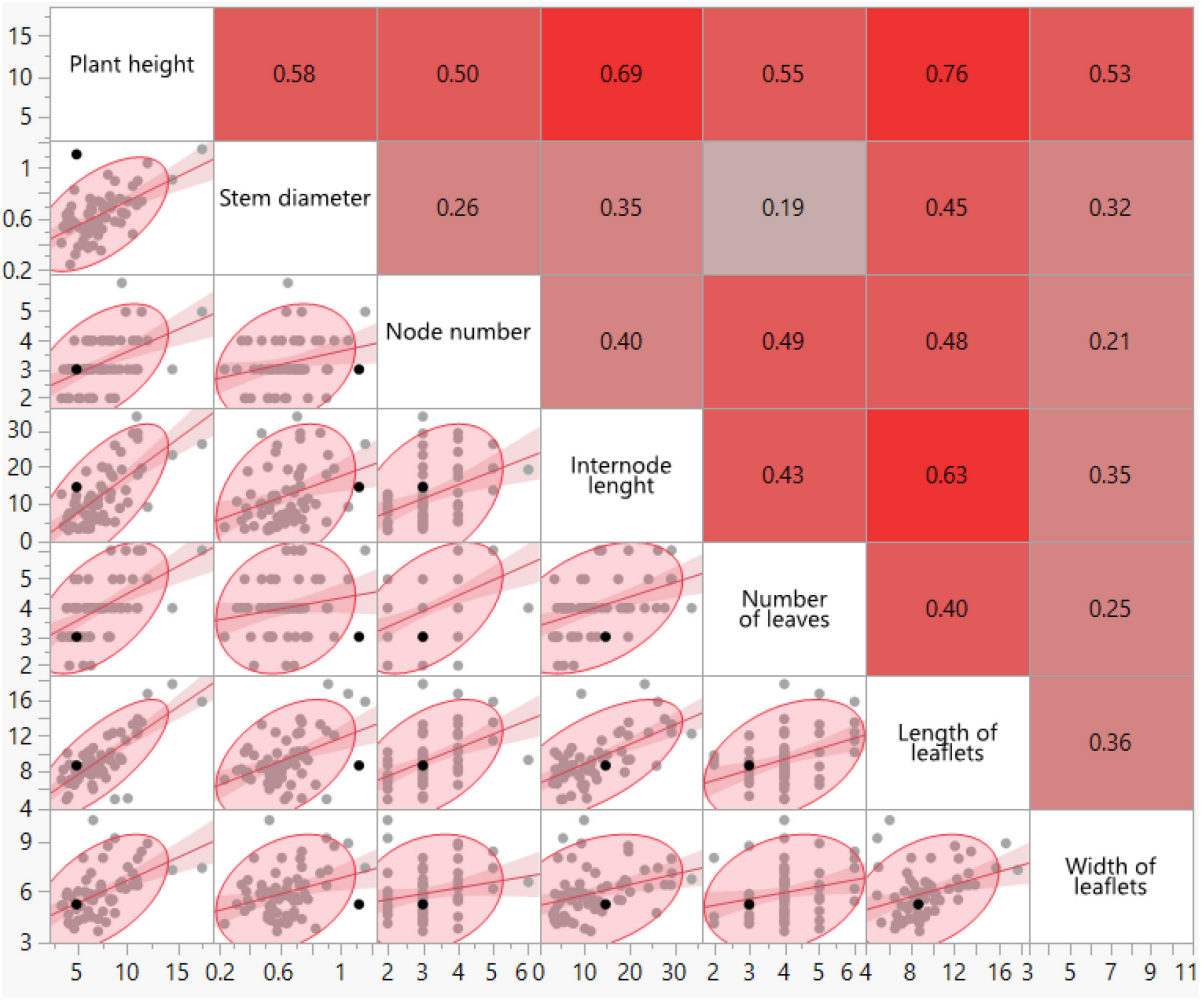
Heatmap graph showing the correlation and significance levels of the characteristics examined in alfalfa plants. (The correlation analysis was performed using the mean values of the 21 Cultivar * Treatment combinations, where each data point represents the average of three biological replicates (n=3). This ensures that the correlation reflects the physiological response of the genotype-treatment interaction, rather than the variance of individual replicates.).

The results of the correlation analysis in **Table 1** (**Figure 2**) show that there are significant relationships between several key morphological traits in alfalfa. Plant height exhibited strong positive correlations with stem diameter (r=0.5771, p<0.0001), internode length (r=0.6918, p<0.0001), number of leaves (r=0.5528, p<0.0001), length of leaflets (r=0.7643, p<0.0001), and width of leaflets (r=0.5262, p<0.0001). Similarly, stem diameter showed significant positive correlations with node number (r=0.2601, p<0.05), length of leaflets (r=0.4502, p<0.001), and width of leaflets (r=0.3193, p<0.01). Node number was positively correlated with internode length (r=0.3951, p<0.01), number of leaves (r=0.4906, p<0.0001), and length of leaflets (r=0.4849, p<0.0001). Internode length also displayed significant positive correlations with number of leaves (r=0.4261, p<0.001), length of leaflets (r=0.6336, p<0.0001), and width of leaflets (r=0.3466, p<0.01). Furthermore, number of leaves was positively correlated with length of leaflets (r=0.4030, p<0.001) and width of leaflets (r=0.2510, p<0.05). Finally, length of leaflets showed a positive correlation with width of leaflets (r=0.3578, p<0.01). These findings suggest a positive correlation observed between these morphological characteristics in alfalfa, where increases in one trait are often associated with increases in others.

### 3.2 Quantitative RT-PCR analysis of salt stress-responsive expression in alfalfa (*Medicago sativa* L.)

The gene expression levels of salt stress-responsive expression (*GR*, *PCS*, *SOD*, and *ZIP*) in alfalfa samples were investigated by qRT-PCR method. *β-Actin* was used as a housekeeping control gene. Magnesium Sulfate and Calcium Chloride (30 mM) treated samples were compared with an untreated plant sample as a negative control (Fold Change: 1) (**Figure 1**).

When the gene expression levels of *GR* salt stress-responsive expression of *Medicago sativa* plant samples treated in seven different cultivars were compared with the negative control; Kaval, Kayseri, Özpınar and Bilensoy (covered and uncovered) cultivars showed reduced expression levels under salt stress conditions in both Magnesium Sulfate and Calcium Chloride (30 mM) applications. Elçi and Sunter cultivar samples showed reduced expression levels under salt stress conditions. When the gene expression levels of *PCS* salt stress-responsive expression of *Medicago sativa* plant samples treated in seven different cultivars were compared with the negative control; no significant differences in *PCS* expression were observed among the cultivars. Kaval and Elçi samples showed response patterns indicating lower levels of effect under Magnesium Sulfate (30 mM) application, whereas Kayseri and Bilensoy (covered) samples exhibited similar response patterns under Calcium Chloride (30 mM) application. Only the Sunter cultivar samples displayed more pronounced responses under both Magnesium Sulfate and Calcium Chloride treatments. Özpınar and Bilensoy (uncovered) cultivar samples showed reduced expression levels under salt stress conditions. When the gene expression levels of *SOD* salt stress-responsive expression of *Medicago sativa* plant samples treated in seven different cultivars were compared with the negative control; Kaval, Kayseri, Elçi, and Sunter cultivars showed reduced expression levels under salt stress conditions in both Magnesium Sulfate and Calcium Chloride (30 mM) applications. Bilensoy (covered and uncovered) and Özpınar cultivar samples may indicate a relatively sensitive response pattern. When the expression levels of *ZIP* salt stress–responsive genes in *Medicago sativa* samples from seven different cultivars were compared with the negative control, no significant or selective expression-reducing effect was observed under either Magnesium Sulfate or Calcium Chloride (30 mM) applications. Under Magnesium Sulfate (30 mM) treatment, Kaval and Kayseri cultivars exhibited expression patterns indicating a reduced-direction effect on *ZIP* gene expression, whereas under Calcium Chloride (30 mM) treatment, a relatively stable expression profile was observed in the Kayseri cultivar, and an expression-reducing trend was detected in the Elçi cultivar. Bilensoy (covered and uncovered), Sunter, and Özpınar cultivar samples exhibited expression patterns indicative of a more pronounced stress-related response (**Figure 1**; **Supplementary Figures 1**–**4**).

According to the results of one-way ANOVA analysis (Dunnett’s multiple comparisons test: diff. among means), significance difference wasn’t found in salt stress-related genes (*GR, PCS, SOD*, and *ZIP*) and cultivars (Kaval, Kayseri, Elçi, Sunter, Bilensoy and Özpınar) compared to the control in *Medicago sativa* samples (1-21) with qRT-PCR.

When comparing these results with field studies conducted in different ecological zones, notable differences appeared. Şeker (2003) reported considerably higher plant heights (ranging from 47.8 to 94.7 cm across cuttings) and stem diameters (ranging from 2.78 to 4.18 mm) for *Medicago sativa* L. varieties Kayseri, ‘CW-3567’, and ‘Planet’ grown under Erzurum’s ecological conditions. Similarly, leaflet dimensions (length: 20.58-28.54 mm; width: 8.58-15.09 mm) observed by Şeker (2003) were substantially larger than those recorded in our greenhouse experiment. These discrepancies likely reflect the influence of environmental factors such as light intensity, soil volume, nutrient availability, and water regime, which are inherently different between controlled pot experiments and open field conditions. Furthermore, the developmental stage at which measurements were taken could also contribute to these variations, as Şeker (2003) data represent measurements from established plants across multiple cuttings over several years.

Yeşil and Şengül (2009) investigated the morphological characteristics of 20 alfalfa ecotypes in a greenhouse setting at Atatürk University. Their reported ranges for plant height (55.80-84.80 cm), stem diameter (1.00-18.90 mm), leaflet length (13.82-18.65 mm), and leaflet width (5.88-9.99 mm) were generally higher than our findings, with the exception of the upper limit of leaflet width which slightly overlaps. The higher values observed by Yeşil and Şengül (2009) could be attributed to differences in the specific ecotypes studied, the duration of their experiment (one year), and potentially larger pot sizes allowing for greater root development and subsequent shoot growth compared to our study. The wider range in stem diameter reported by Yeşil and Şengül (2009) is particularly noteworthy and might indicate significant genetic variability among the studied ecotypes for this trait.

Gökalp et al. (2017) examined the agronomic characteristics of five alfalfa cultivars under Tokat-Kazova ecological conditions over two years. Their reported plant height values (78.1-85.72 cm) and stem diameters (3.19-3.32 mm) were considerably greater than those observed in our greenhouse trial. This further emphasizes the impact of field conditions on plant growth and development. Gökalp et al. (2017) also reported higher main stem numbers per plant (7.9-9.4), which contrasts with the lower node and leaf counts in our early-stage greenhouse plants. The differences in yield parameters reported by Gökalp et al. (2017) are not directly comparable to our study, as we focused on early vegetative growth characteristics rather than biomass production.

Nehir (2017) investigated 51 alfalfa populations collected from Ahi Evran University. Their findings for plant height (96.7 cm), stem diameter (3.3 mm), leaflet length (19.7 mm), and leaflet width (6.5 mm) in field conditions were also generally higher than our greenhouse results, reinforcing the influence of environmental factors on these morphological traits. The significantly higher main stem number per plant (37.2) reported by Nehir (2017) likely reflects the more mature developmental stage and potentially different growth habits of the studied populations compared to the young seedlings in our pot experiment.

Sayar et al. (2019) focused on annual *Medicago* species under field conditions in the Southeastern Anatolia Region. Their reported plant heights (21.27-28.66 cm) were higher than our alfalfa results, which is expected given they studied different species known for potentially distinct growth habits. Their leaflet dimensions (length: 9.43-13.56 mm; width: 8.99-14.49 mm) showed some overlap with our findings, particularly in leaflet length. However, direct comparisons should be made cautiously due to the different species and the fact that Sayar et al. (2019) focused on mature plants in field conditions.

In conclusion, while our greenhouse pot experiment provides valuable initial data on the early growth characteristics of the studied alfalfa cultivars under controlled conditions, the significantly higher values reported in field studies by Şeker (2003); Gökalp et al. (2017), and Nehir (2017), as well as the greenhouse study by Yeşil and Şengül (2009) with different ecotypes, highlight the substantial impact of environmental factors and plant developmental stage on morphological traits. Further research under field conditions in relevant ecological zones is crucial to fully evaluate the agronomic potential of these alfalfa cultivars. The differences observed also underscore the importance of considering the specific experimental conditions when comparing results across different studies.

The integrated morphological analysis of seven alfalfa cultivars from Türkiye under Magnesium Sulfate and Calcium Chloride treatments revealed significant cultivar- and treatment-dependent variations across all measured traits. Notably, traits such as node number, internode length, and leaflet dimensions exhibited statistically significant interactions between genotype and salt source, indicating genotype-specific responses to salinity. While overall salinity stress led to reduced vegetative growth, certain cultivars, such as Elçi and Bilensoy-uncovered, exhibited response patterns associated with an expression-reducing effect on stress-responsive genes. Moreover, strong positive correlations among plant height, internode length, leaflet size, and leaf number suggest a coordinated morphological response to salt stress. These findings underscore the potential for selecting or managing specific cultivars and nutrient treatments to mitigate salinity effects and improve early-stage alfalfa growth under saline conditions.

Studies examining the potential response pattern mechanisms of the expression of the salt stress-related *GR*, *PCS, SOD* and *ZIP* genes specific to *Medicago* plants are not sufficient in the literature. Studies addressing the gene expression related to salt stress in these plants are quite limited. Li et al. (2022a) investigated antioxidant enzyme activities and proline accumulation in two alfalfa cultivars exposed to NaCl stress and reported no significant differences in *SOD, CAT, GR*, and *APX* activities between cultivars under control conditions. Following salt treatment, *SOD, CAT*, and *GR* activities increased markedly in both roots and shoots, with generally higher enzyme activities observed in the Magnum Salt cultivar compared to Adrenalin. Notably, *GR* activity in shoots did not differ significantly between cultivars, indicating tissue-specific regulation of antioxidant responses under monovalent salinity stress. In contrast to these enzyme activity–based observations, the present study examined gene expression–level responses of *GR* and *SOD* under divalent salt stress conditions (30 mM MgSO_4_ and CaCl_2_) and evaluated alfalfa plants as a whole (root and shoot combined). Our results revealed cultivar-specific reductions in *GR* transcript levels rather than induction, suggesting that Mg²^+^ and Ca²^+^-based salinity may trigger distinct transcriptional regulatory mechanisms compared to NaCl stress. Specifically, under Magnesium Sulfate treatment, *GR* gene expression decreased approximately 1-fold in Kaval, Kayseri, Özpınar, and Bilensoy (covered) cultivars and 0.8-fold in Bilensoy (uncovered). Under Calcium Chloride treatment, *GR* expression levels decreased in Kaval and Özpınar (0.7-fold), Kayseri (0.8-fold), and Bilensoy cultivars (0.9-fold in covered and 1-fold in uncovered plants). The contrasting patterns between increased *GR* enzyme activity reported by Li et al. (2022a) and reduced *GR* transcript levels observed in our study highlight the complexity of antioxidant regulation in alfalfa. These differences may reflect salt-type–specific signaling pathways, differential ion toxicity (monovalent vs. divalent cations), and distinct regulatory layers governing enzyme activity and gene expression. Taken together, these findings suggest that GR regulation may be context-dependent and that transcriptional downregulation of GR, in association with morphological response patterns, may represent an adaptive adjustment of redox-related pathways rather than a universal enhancement of antioxidant enzyme activity.

Salt stress tolerance and antioxidant activities of transgenic *Medicago sativa* plants were determined in Ma et al., 2023b. The expression of reactive oxygen species (ROS) scavenging genes was determined in alfalfa under control and salt treatment. In addition, salt stress-related *SOD, POD* and *CAT* antioxidant enzymatic expressions were investigated and an increase was found compared to the control.

In our study, salt stress-related *SOD* gene expression of treatments applied to natural alfalfa samples was determined. The gene expression levels of *SOD* salt stress-responsive expression of *Medicago sativa* samples applied with Magnesium Sulfate decreased in Kaval (1-fold), Kayseri (0.8-fold), Elçi (0.6-fold), and Sunter (0.3-fold). The gene expression levels decreased in Calcium Chloride applications in Kaval (0.9-fold), Kayseri (0.9-fold), Elçi (0.5-fold), and Sunter (0.8-fold).

The activities of antioxidant enzymes such as *SOD* and *POD* in the early flowering period of alfalfa were measured by Hou et al., 2022, and the decrease in soil salinity was associated with the increase in the amount of applied irrigation. When salt was more than 3g/kg, *POD* activity increased in alfalfa, while *SOD* activity decreased, and stress resistance was increased. When salt was less than 3g/kg, *SOD* and *POD* activities increased in alfalfa. Moderate salt levels are consistent with stress tolerance.

According to the data of our study, *SOD* activity decreased in four different cultivars following the application of Magnesium Sulfate and Calcium Chloride at 30 mM, indicating a reduced activity–directed response under salt stress conditions.

According to Guiza et al., 2022 study, the enzyme activity differs after sodium chloride (150 mM) treatment applied to Alfalfa (*Medicago sativa* L.) samples. While *SOD* activity increased in two cultivars, it was shown that the other five cultivars were more sensitive to sodium chloride than the others. It was stated that *CAT* activity exhibited patterns similar to those of *SOD* activity, and both were associated with gene expression–reducing effects observed in the same cultivars.

Our findings are consistent with Guiza et al., 2022 study, and salt stress tolerance was determined in two cultivars and one gene activity. While *PCS* was downregulated only in Bilensoy and Özpınar cultivars, salt stress sensitivity was detected in *ZIP* activity in all cultivars. The gene expression levels of *PCS* salt stress-responsive expression of *Medicago sativa* samples applied with Magnesium Sulfate decreased in Kaval (0.8-fold), Elçi (0.5-fold), and Sunter (0.7-fold). The gene expression levels decreased in Kayseri (0.9-fold), Sunter (0.1-fold), and Bilensoy (covert, 0.8-fold) in Calcium Chloride applications.

In the results of Pacheco-Insausti et al., 2023, inoculated alfalfa (*Medicago sativa*) plants were exposed to salinity and cadmium together. To evaluate stress responses, *SOD* and *CAT* enzymatic activities and gene expression analyses and *PCS* activity were determined. The activity of the tolerance mechanism was indicated by the increase in *SOD* and *CAT* due to NaCl and CdCl_2_ stress. In addition, the decrease in *PCS* gene expression showed that the plants could withstand the combined stress. We found that *PCS* gene expression was tolerated in alfalfa plants exposed to stress outside the two cultivars.

Rahman et al., 2020, investigated the recovery of alfalfa plants treated with Mercury (Hg) with *GSH* supplementation. While photosynthesis, which decreased with Hg increase, was shown to increase again with *GSH* application, upregulation was also determined in *GSH* and *PCS* genes. In addition, it was found that oxidative damage caused by Hg-induced H_2_O_2_ and lipid peroxidation stimulated *GR* activity.

Alfalfa plants were exposed to MgSO_4_ and CaCl_2_ salinity stress, which resulted in decreased *GR, SOD*, and *PCS* gene expression levels in specific cultivars. This response pattern may be interpreted as an expression-reducing effect under saline conditions (30 mM), suggesting a potential modulation of stress-related pathways that could influence plant performance under saline growing conditions.

Our results showed that MgSO_4_ and CaCl_2_ treatments at 30 mM increased salt stress–related *PCS* and *ZIP* gene expression, and that Özpınar and Bilensoy cultivars exhibited more pronounced transcriptional responses compared to the other cultivars. Li et al., in 2022b review article, discussed the roles of *ZIP* proteins in plant response to abiotic stresses in *Medicago* species and indicated potential targets for stress tolerance. In their study, they explained that the *ZIP* family of *Medicago* plants plays a role in regulating transcription factors and induces abiotic stress-related *TF* expression.

In our study, we provided more extensive and detailed information about *Medicago sativa* species and evaluated the expressions of *ZIP* mRNA related to salinity stress in six different cultivars and two different treatments. The gene expression levels of *ZIP* salt stress-responsive expression of *Medicago sativa* samples applied with Magnesium Sulfate decreased in Kaval (0.2-fold) and Kayseri (0.4-fold). In Calcium Chloride applications, the gene expression levels decreased only in Elçi (0.4-fold). These cultivar- and salt-type–dependent differences in *ZIP* expression may reflect genotype-specific transcriptional regulation and differential ionic or signaling responses triggered by divalent salts.

There may be several main reasons why salt stress applications with both MgSO_4_ and CaCl_2_ salts give different results in *Medicago* (clover) type cultivar samples. One of these is genetic factors such as root structure, osmotic balance protection mechanisms, ability to limit salt entry into the cell, and strength of antioxidant defense systems. Another is that MgSO_4_ and CaCl_2_ applications may have different effects on plant physiology due to the different ions they contain, and these ions may interact with other elements in the soil.

One-way ANOVA analysis (Dunnett’s multiple comparisons test: difference between means) was performed on both salt stress-related genes (*GR*, *PCS*, *SOD*, and *ZIP*) and cultivars (Kaval, Kayseri, Elçi, Sunter, Bilensoy, and Özpınar) in *Medicago sativa* samples (1–21) compared to the control by qRT-PCR. However, no statistically significant differences were detected according to the results. Despite this, the integrated evaluation of morphological traits and salinity stress-related gene expression revealed biologically meaningful response trends across cultivars. The consistent downregulation of *GR* and *PCS* observed in ‘Kaval’ and ‘Kayseri’ under both MgSO_4_ and CaCl_2_ treatments suggests potential genotype-dependent transcriptional adjustments contributing to ionic and redox homeostasis. These trends, although not statistically significant due to biological variability and sample size limitations, may still reflect underlying physiological adaptations to salt stress. Moreover, morphological parameters such as node number, internode length, and leaflet size showed significant genotype-by-treatment interactions, aligning with gene expression tendencies. This correspondence between molecular and phenotypic responses highlights that even statistically nonsignificant expression patterns can provide insight into adaptive tolerance mechanisms under salt stress. Future studies incorporating larger biological replicates and multivariate models are warranted to validate these preliminary yet biologically relevant findings.

The observed alignment between molecular and phenotypic responses supports the presence of regulatory response patterns associated with gene expression–reducing effects under stress conditions. For instance, the downregulation of *GR* in Kaval, Kayseri, Özpınar, and Bilensoy (which were the most sensitive cultivars morphologically, showing the greatest reductions in growth traits) correlates strongly and significantly with physical reductions. The high correlation coefficient (r = 0.81) observed between *GR* expression and traits such as internode length, despite the lack of statistical significance in individual transcriptional changes, a notable associative trend. It suggests that even subtle, non-significant modulations in the redox-regulating genes can track consistently with phenotypic shifts across diverse genotypes. This implies that the cumulative effect of minor transcriptional adjustments may be phenotypically relevant, even if they do not meet the threshold of statistical significance in a single-point ANOVA.

In this context, the observed down-regulation of genes should not be interpreted as direct evidence of salt stress potential response pattern/tolerance. Rather, these transcriptional patterns may also reflect suppressed defense responses, early stress sensitivity, or cultivar-specific transcriptional timing. Our study presents molecular observations on the effects of magnesium sulfate and calcium chloride treatments on salt stress–related gene expression in alfalfa plants. Rather than definitive conclusions, these findings are interpreted as preliminary insights describing potential transcriptional response patterns among different cultivars under salt stress, which may contribute to a better understanding of stress-associated regulatory processes relevant to plant growth conditions.

## 4 Conclusion

In our study, seven different cultivars (*Medicago sativa* L.) from Türkiye (Kaval, Kayseri, Elçi, Sunter, Özpınar, Bilensoy-Covered, Bilensoy-Uncovered) were treated with Magnesium Sulfate and Calcium Chloride (30 mM). qRT-PCR analysis was performed to determine the expression of related genes (*GR, PCS, SOD* and *ZIP*) after salt stress application.

This study provides new insights into the expression patterns of four salinity stress-related genes (*GR, PCS, SOD*, and *ZIP*) in seven cultivars of *Medicago sativa* treated with magnesium sulfate and calcium chloride (30 mM). In this study, seven different *Medicago sativa* L. cultivars were evaluated under Magnesium Sulfate and Calcium Chloride treatments through an integrated analysis of morphological traits and gene expression. Significant differences were observed in morphological parameters—particularly node number, internode length, and leaflet size—indicating strong genotype-by-treatment interactions. Although no statistically significant differences were detected in gene expression levels according to one-way ANOVA analysis (Dunnett’s multiple comparisons test), observable trends were identified—particularly in the cultivars Kaval and Kayseri—indicating genotype-dependent transcriptional responses. Morphological analyses revealed notable variations among cultivars, especially in internode length and leaflet size, suggesting differential physiological adaptability under salt stress. These changes were particularly notable in the Kaval, Kayseri, Özpınar, and Bilensoy cultivars, which exhibited observable trends of decreased *GR* gene expression.

Although these differences were not statistically significant, the observed gene expression patterns showed qualitative alignment with the morphological responses to salinity stress. Accordingly, these results are interpreted not as definitive evidence, but as preliminary observations describing potential genotype-specific transcriptional response tendencies under salt stress conditions.

Within this framework, the findings may offer descriptive insights relevant to alfalfa breeding and cultivar selection for saline soils by highlighting cultivar-dependent response patterns rather than confirmed tolerance traits. Moreover, when considered alongside morphological performance, these molecular observations provide a preliminary basis for identifying candidate genotypes for further evaluation in stress physiology studies and forage production systems under saline field conditions.

The identified associations between leaflet size and *GR* expression may potentially serve as preliminary indicators for selection for screening salt-tolerant alfalfa germplasm. Specifically, cultivars like ‘Elçi’ and ‘Sunter’, which maintained relatively stable growth parameters, could be utilized as genetic donors in breeding programs aimed at developing resilience against divalent cation-dominant salinity.

## Data Availability

The datasets presented in this study can be found in online repositories. The names of the repository/repositories and accession number(s) can be found in the article/**Supplementary Material**.
