# Supplementary material for: Integrated analysis of morphological traits and salinity stress-related gene expression in alfalfa (Medicago sativa L.) from Türkiye under magnesium sulfate and calcium chloride treatments
Source: Front Plant Sci. 2026 May 7;17:1764060. doi: 10.3389/fpls.2026.1764060 (PMC13189781; doi:10.3389/fpls.2026.1764060)
Supplement: Supplementary file 1 [file SupplementaryFile1.docx]

**SUPPLEMENTARY FILE**

**Integrated Analysis of Morphological Traits and Salinity Stress-Related Gene Expression in Alfalfa (Medicago sativa L.) from Türkiye under Magnesium Sulfate and Calcium Chloride Treatments**

Muhammed İkbal Çatal^1,^ Seda Mesci^2,3^*

^1^ Recep Tayyip Erdoğan University, Faculty of Agriculture, Department of Field Crops, Rize, Türkiye.

^2^ Project Coordination and Guidance Office, Rectorate, Hitit University, Çorum, Türkiye

^3^ Food Safety, Agricultural Application and Research Center, Hitit University, Çorum, Turkey

* Corresponding author

Phone: +90 364 2192892, e-mail: [sedamesci@hitit.edu.tr](mailto:sedamesci@hitit.edu.tr), ORCID: 0000-0002-5440-302X

**CONTENTS**

**Figure S1.** qRT-PCR mRNA gene expression levels of the *GR* gene in ***Medicago sativa*** samples (1-21), Control: untreated..……………………………………………………….……………………...3

**Figure S2.** qRT-PCR mRNA gene expression levels of the *PCS* gene in ***Medicago sativa*** samples (1-21), Control: untreated..……………………………………………………….……………………...3

**Figure S3.** qRT-PCR mRNA gene expression levels of the *SOD* gene in ***Medicago sativa*** samples (1-21), Control: untreated..…………………………………………………....……………………...4

**Figure S4.** qRT-PCR mRNA gene expression levels of the *ZIP* gene in ***Medicago sativa*** samples (1-21), Control: untreated..……………………………………………………….……………………...4

**
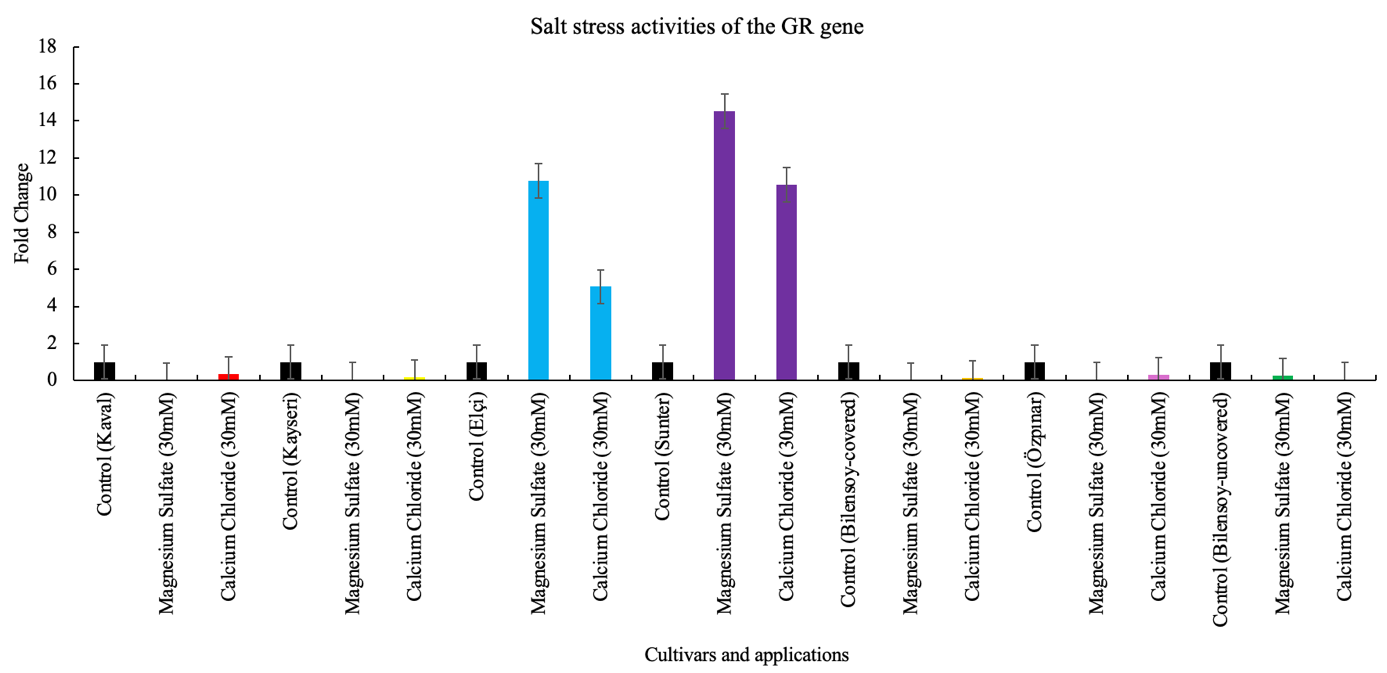
Figure S1.** qRT-PCR mRNA gene expression levels of the *GR* gene in ***Medicago sativa*** samples (1-21), Control: untreated

**
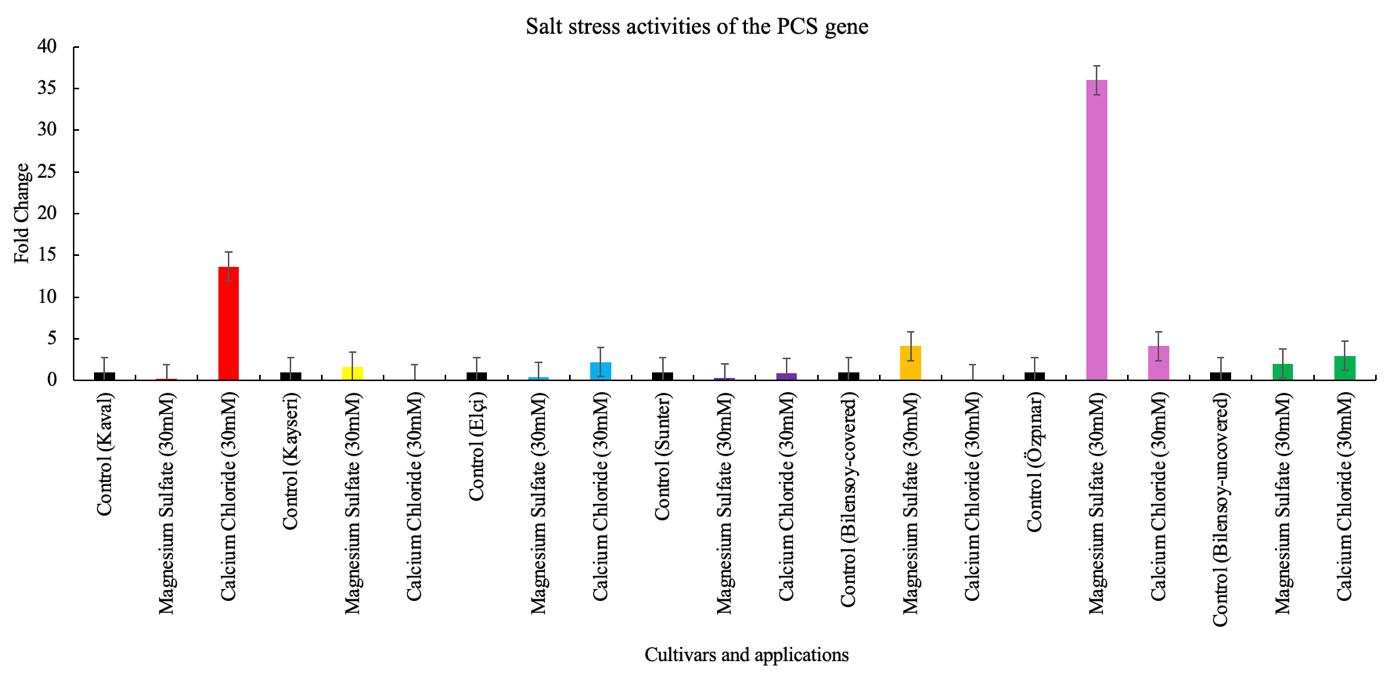
Figure S2.** qRT-PCR mRNA gene expression levels of the *PCS* gene in ***Medicago sativa*** samples (1-21), Control: untreated

**
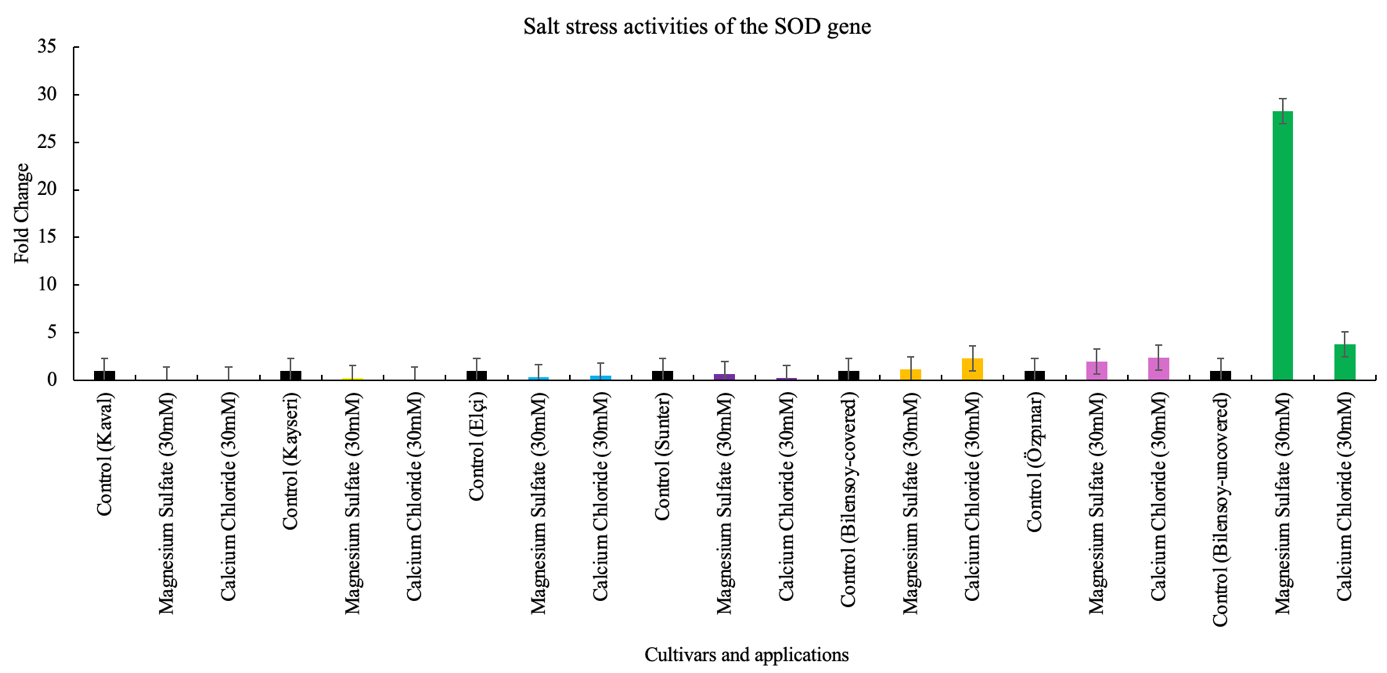
Figure S3.** qRT-PCR mRNA gene expression levels of the *SOD* gene in ***Medicago sativa*** samples (1-21), Control: untreated


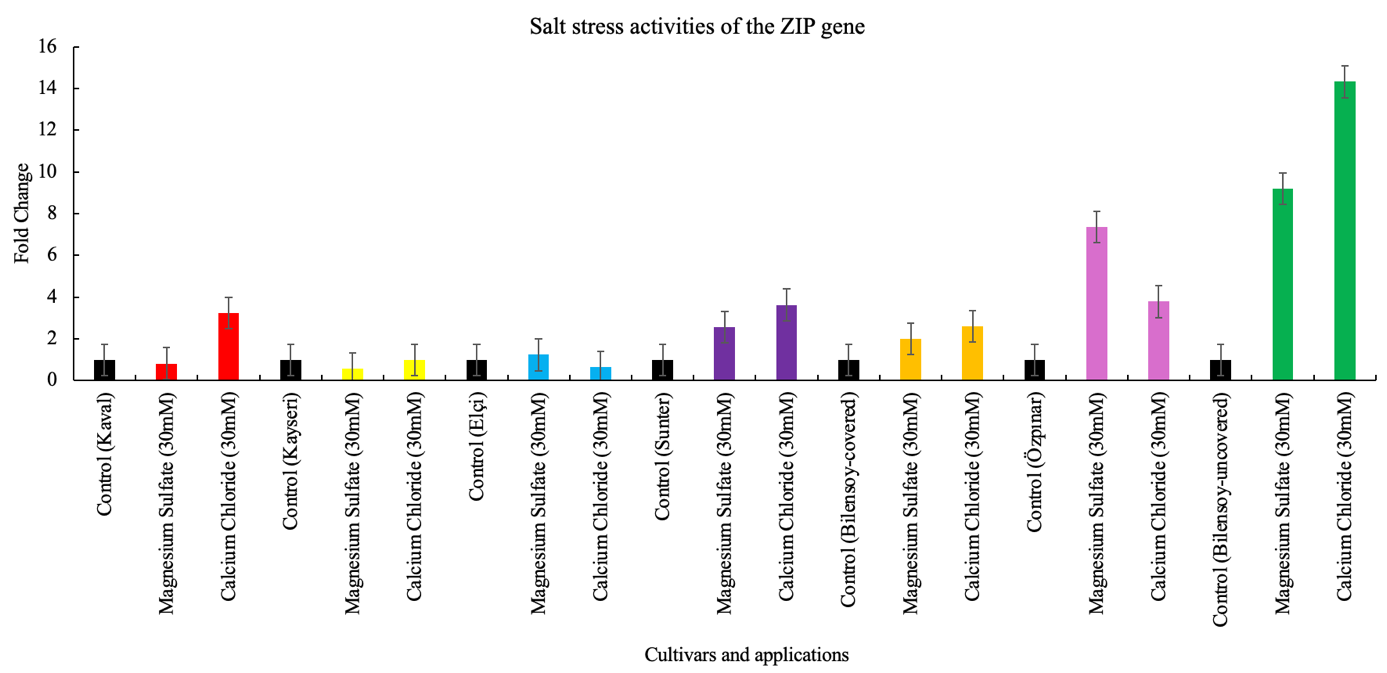
**Figure S4.** qRT-PCR mRNA gene expression levels of the *ZIP* gene in ***Medicago sativa*** samples (1-21), Control: untreated
